# Molecular mechanisms of cell death in neurological diseases

**DOI:** 10.1038/s41418-021-00814-y

**Published:** 2021-06-07

**Authors:** Diane Moujalled, Andreas Strasser, Jeffrey R. Liddell

**Affiliations:** 1 The Walter and Eliza Hall Institute, Parkville, VIC Australia; 2 Department of Medical Biology, The University of Melbourne, Parkville, VIC Australia; 3 Department of Biochemistry and Pharmacology, The University of Melbourne, Parkville, VIC Australia

**Keywords:** CNS cancer, Neuroscience

## Abstract

Tightly orchestrated programmed cell death (PCD) signalling events occur during normal neuronal development in a spatially and temporally restricted manner to establish the neural architecture and shaping the CNS. Abnormalities in PCD signalling cascades, such as apoptosis, necroptosis, pyroptosis, ferroptosis, and cell death associated with autophagy as well as in unprogrammed necrosis can be observed in the pathogenesis of various neurological diseases. These cell deaths can be activated in response to various forms of cellular stress (exerted by intracellular or extracellular stimuli) and inflammatory processes. Aberrant activation of PCD pathways is a common feature in neurodegenerative diseases, such as amyotrophic lateral sclerosis (ALS), Alzheimer’s disease, Parkinson’s disease, and Huntington’s disease, resulting in unwanted loss of neuronal cells and function. Conversely, inactivation of PCD is thought to contribute to the development of brain cancers and to impact their response to therapy. For many neurodegenerative diseases and brain cancers current treatment strategies have only modest effect, engendering the need for investigations into the origins of these diseases. With many diseases of the brain displaying aberrations in PCD pathways, it appears that agents that can either inhibit or induce PCD may be critical components of future therapeutic strategies. The development of such therapies will have to be guided by preclinical studies in animal models that faithfully mimic the human disease. In this review, we briefly describe PCD and unprogrammed cell death processes and the roles they play in contributing to neurodegenerative diseases or tumorigenesis in the brain. We also discuss the interplay between distinct cell death signalling cascades and disease pathogenesis and describe pharmacological agents targeting key players in the cell death signalling pathways that have progressed through to clinical trials.

## Introduction

Programmed cell death (PCD) is required for normal development and maintenance of tissue homoeostasis, and the elimination of damaged, infected or obsolete cells in multicellular organisms. Seminal discoveries by Kerr et al. in 1972 identified the hallmark ultrastructural features of cells undergoing programmed suicide, where the term ‘apoptosis’ was coined for this form of PCD [1]. These features include cytoplasmic shrinkage, nuclear condensation and fragmentation and the formation of apoptotic bodies that are evident in various tissues under physiological or certain pathological conditions. The family of anti- and pro-apoptotic B cell lymphoma-2 (BCL-2) protein family members has been discovered that regulate this pathway and are comprised of three subgroups based on their structure and function with the presence of conserved regions termed BCL-2 homology (BH) motifs). This includes the anti-apoptotic proteins (BCL-2, BCL-XL, MCL-1, BCL-W and A1/BFL1), the BH3-only proteins (BIM, PUMA, BID, BMF, BAD, HRK, BIK, NOXA), the critical initiators of apoptosis and multi-BH domain proteins (BAX and BAK), the essential effectors of apoptosis that form oligomers that cause mitochondrial outer membrane permeabilisation (MOMP), thereby releasing apoptogenic factors that promote a cascade of caspase (aspartate-specific cysteine proteases) activation [2, 3]. Upon activation, caspases cleave hundreds of cellular substrates, thereby precipitating the morphological features of apoptosis and demolition of the cell [4–6].

Normal development and tissue homoeostasis in multicellular organisms depend on orchestrated PCD signalling events that are tightly regulated. During embryogenesis, the elimination of cells by PCD is necessary for adequate moulding of certain tissues, for example the sculpting of the digits of vertebrate limbs [7]. The central nervous system (CNS), comprised of the brain and spinal cord, is shaped by PCD where signalling events that are tightly regulated at a temporal and spatial level result in establishment of the neural architecture. In normal neural embryonic and post-natal development, apoptosis is the major form of PCD. Apoptosis can affect distinct cell populations, including neural precursor cells (NPCs), differentiated post-mitotic neurons and glial cells, ensuring the survival only of cells that are of the correct size and shape and have made the proper connections with their axons and neurites [8]. In mouse embryos, neurogenesis occurs as early as E12 when NPCs exit the cell cycle and differentiate into post-mitotic neurons. It was shown that the anti-apoptotic BCL-2 family members myeloid cell leukaemia-1 (MCL-1) and BCL-2-related gene long isoform (BCL-XL) play critical roles in cell survival during developmental neurogenesis. Neuronal-specific ablation of both proteins resulted in massive apoptotic cell death throughout the entire CNS [9], and even loss of either gene caused fatal defects in the brain [10, 11]. Conversely, the combined absence of BAX, BAK (and their relative BOK) causes an increase of neurons within certain areas of the brain [12], although the impact of this for brain function and behaviour is not known. The critical opposing roles of the pro- and anti-apoptotic members of the BCL-2 family are demonstrated by the observation that the combined loss of one allele of *Mcl-1* and one allele of *Bclx* (*Mcl1*^*+/−*^*Bclx*^*+/−*^ mice) causes severe craniofacial abnormalities and early post-natal death, while additional loss of one allele of *Bim* (*Mcl1*^*+/−*^*Bclx*^*+/−*^*Bim*^*+/−*^ mice) prevents these abnormalities completely [13].

While removal of superfluous neuronal cells is vital for normal brain function, aberrant death of distinct neuronal cell populations is a hallmark of pathology associated with neurodegenerative diseases, such as ALS, Alzheimer’s disease (AD), Parkinson’s disease (PD) and Huntington’s disease (HD) (reviewed in [14]). Conversely, defects in PCD of neuronal cells or other cell types in the brain is thought to promote development of brain cancers, such as the highly aggressive glioblastoma multiforme [15]. The cell death pathways are associated with distinct morphological and biochemical features (refer to Table 1 for characteristic morphological and biochemical hallmarks, highlighting fundamental differences in the pathways). For example, apoptosis is typically associated with cell shrinkage, while necroptosis involves cell swelling and leakage of cellular contents.

**Table 1:** Cell death pathways and associated morphological and biochemical hallmark features.

Cell death pathway	Morphological features and key biochemical pathway components	References
Apoptosis	Nuclear fragmentation, plasma membrane blebbing, cell shrinkage (pyknosis), formation of apoptotic bodies and phagocytosis by neighbouring cells. Pro-apoptotic BCL-2 family members, caspase activation, cleavage of hundreds of caspase substrates (e.g. ICAD, PARP), PS exposure, ΔΨm dissipation, MOMP and ROS production.	[1, 21, 150]
Necroptosis	Cytoplasmic swelling (oncosis), loss of plasma membrane integrity, swelling of cytoplasmic organelles. RIPK1, RIPK3, MLKL, phosphorylation and ubiquitination of RIPK1, formation of the necrosome complex in the cytosol, phosphorylation and activation of MLKL, the effector of caspases, ROS production and release of DAMPs (and in infected cells also PAMPs).	[57–60]
Autophagy	Accumulation of autophagic vacuoles, vacuolisation of the cytoplasm, no chromatin condensation. *atg* family of gene encoded proteins, LC3-I to LC3-II conversion and cleavage of p62.	[82, 151]
Ferroptosis	Smaller mitochondria with decreased cristae, increased density and rupture of mitochondrial membrane but with normal nucleus. Iron accumulation, cysteine deprivation and/or glutathione peroxidase inactivation culminating in lipid peroxidation.	[102]
Pyroptosis	Rupture of the plasma membrane and lack of cell swelling. Inflammatory induced activation of the initiator caspases, caspase-1 and -11, and consequent activation of the effector caspases, caspase-3 and -1. Release of bio-active IL-1β and IL-18 and proteolytic activation of GSDMD, the essential effector of pyroptosis.	[135, 136]
Necrosis	Plasma membrane rupture, swelling of cytoplasmic organelles, lack of inter-nucleosomal DNA fragmentation, depletion of ATP, involvement of calpains and cathepsins, release of DAMPs (and in infected cells also PAMPs).	[139, 152]

*ΔΨm* mitochondrial transmembrane potential, *MMP* mitochondrial membrane potential, *LC3* microtubule-associated protein light chain 3, *ROS* reactive oxygen species, *PARP1* poly ADP-ribose polymerase 1, *PS* phosphatidyl serine, *GSDMD* gasdermin D, *IL-1β* interleukin-1β*,* *ATP* adenosine triphosphate, *calpains* calcium-activated non-lysosomal proteases.

The aetiology of neurodegenerative diseases is multifactorial, being associated with defects in different cellular processes, such as response to oxidative stress, excitotoxicity, mitochondrial dysfunction, protein misfolding (ER stress) and inflammation [16–19]. Considerable evidence supports a role of cell death in the pathogenesis of various diseases of the brain and peripheral nervous system. Nevertheless, an important question remains whether defects in cell death signalling and neuronal cell death is a primary or only a secondary response to the insults that cause these diseases, and how different PCD pathways and additional processes interact to cause the demise of neuronal cells and other cell types in these pathologies.

In this review, we provide a brief overview of PCD pathways, namely apoptosis, necroptosis, pyroptosis, ferroptosis as well as cell death associated with autophagy and unprogrammed necrosis. We discuss their known and proposed roles in the pathogenesis of neurodegenerative diseases and brain cancer, such as GBM. We also describe already existing and proposed therapeutic strategies to target central regulators of the various PCD pathways for the treatment of neurological diseases.

## Programmed cell death signalling pathways and their roles in neurological diseases

### Apoptosis

**Fig. 1:**
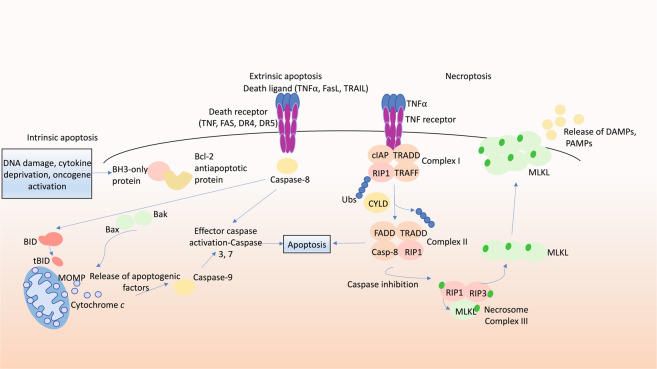
Molecular pathways of apoptosis and necroptosis. Intrinsic apoptosis signalling: in response to growth factor deprivation, DNA damage or oncogene activation, BH3-only pro-apoptotic proteins are induced transcriptionally or post-transcriptionally. The BH3-only proteins bind to and inhibit the anti-apoptotic BCL-2 proteins. This leads to release and activation of the effectors of cell death, BAX and BAK, which then oligomerise and promote mitochondrial outer membrane permeabilisation (MOMP), leading to release of apoptogenic factors, including cytochrome *c* and Smac/Diablo. This initiates a cascade of caspase activation, cleavage of hundreds of cellular proteins and consequent cell demolition. Death receptor induced (extrinsic) apoptosis signalling: stimulation of death receptors (members of the TNFR family with an intracellular death domain) by their cognate ligands (e.g. stimulation of FAS by FASL) results in adaptor protein (FADD, TRADD) mediated recruitment and activation of the initiator caspase, caspase-8 (in humans also caspase-10), which can then activate downstream effector caspases (caspases-3, -7), resulting in cell demolition (see above). The death receptor induced apoptosis pathway can connect to the intrinsic apoptotic pathway by proteolytic activation of the pro-apoptotic BH3-only protein BID (to generate tBID) by caspase-8. Necroptosis: the best characterised pathway triggering necroptosis is via TNFR1 stimulation. Upon binding of TNFα to TNFR1, cIAPs, RIPK1, TRAFs and TRADD are recruited to the intracellular part of TNFR1, forming the TNFR1 signalling complex I, which activates NFkB and AP1 transcription factors and thereby stimulates cell survival and proliferation. Upon deubiquitylation of RIPK1 by CYLD, RIPK1 can bind to TRADD, FADD and caspase-8 forming complex II which can drive caspase-8 mediated apoptosis (see above). In the absence of caspase-8 (genetic ablation or pharmacological inhibition) and the absence or inhibition of cIAP1/2, the necrosome (complex III) is formed where RIPK3 can phosphorylate MLKL, promoting its translocation to the plasma membrane where MLKL causes cell lysis, resulting in lytic cell death with release of DAMPs and PAMPs.

**Fig. 2:**
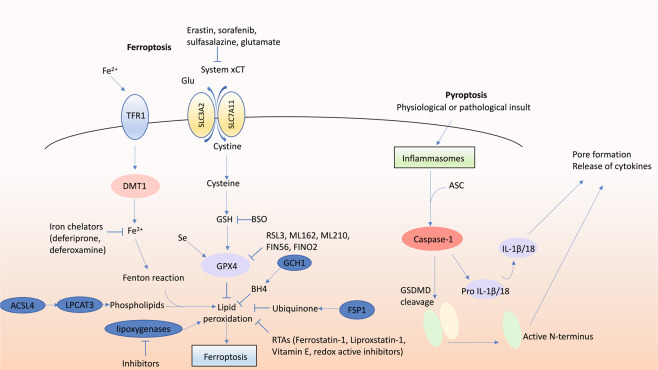
Molecular pathways of ferroptosis and pyroptosis. Ferroptosis is potentiated by iron which can be imported by TFR1 and DMT1, and requires the presence of susceptible arachidonic acid-containing phospholipids generated by ACSL4 and LPCAT3. Ferroptosis is endogenously inhibited by GPX4 which requires GSH as a substrate, by ubiquinone which is reduced by FSP1, by BH4 which is generated by GCH1, and by other endogenous RTAs such as vitamin E. Ferroptosis is promoted by disruption of cysteine supply via inhibition of the glutamate/cystine antiporter system xCT (by compounds such as erastin, sorafenib, sulfasalazine or glutamate), or impaired GPX4 activity due to insufficient glutathione or selenium or by direct inhibition (e.g. RSL3, ML162, ML210, FIN56, FINO2). Ferroptosis inhibitors include iron chelators (such as deferiprone and deferoxamine), RTAs (including ferrostatin-1 and liproxstatin-1) and lipoxygenase inhibitors. Pyroptosis can be activated in response to physiological or pathological insults which result in activation of inflammasomes, such as the NLRP3 inflammasome, in which the adaptor ASC is recruited, resulting in activation of caspase-1. Caspase-1 proteolytically processes pro-IL-1β and pro-IL-18 into the bio-active forms of these cytokines. Caspase-1 can also cleave GSDMD, and the N‐terminal fragment of GSDMD is recruited to the plasma membrane where it causes pore formation, cell swelling and plasma membrane rupture. BH4 tetrahydrobiopterin, BSO Buthionine sulfoximine, DMT1 divalent metal transporter 1, GCH1 GTP cyclohydrolase-1, GPX4 glutathione peroxidase 4, GSH glutathione, RTAs radical trapping antioxidants, Se selenium, System xCT glutamate/cystine antiporter, TFR1 transferrin receptor 1, NLRP3 NLR family pyrin domain‐containing 3.

Apoptosis can be triggered by two distinct pathways: the intrinsic (also called mitochondrial or BCL-2-regulated) pathway and the death receptor (also called extrinsic) pathways [20, 21]. The intrinsic pathway is regulated by the pro- and anti-apoptotic members of the BCL-2 protein family. In healthy cells the anti-apoptotic proteins BCL-2, BCL-XL, MCL-1, BCL-W and A1/BFL1 safeguard cell survival by restraining the essential effectors of cell death BAX and BAK [2]. In response to intracellular stress (e.g. growth factor deprivation, DNA damage, ER stress), the BH3-only proteins (BIM, PUMA, BID, BMF, BAD, HRK, BIK, NOXA), the critical initiators of apoptosis, are transcriptionally or post-transcriptionally upregulated [22]. The BH3-only proteins bind with high affinity to anti-apoptotic BCL-2 proteins, thereby liberating BAX and BAK. Some BH3-only proteins are reported to also activate BAX and BAK directly [2, 3, 23]. Upon activation, BAX and BAK form oligomers that cause MOMP with consequent mitochondrial release of cytochrome c and Smac/DIABLO [21]. These apoptogenic factors promote activation of the caspase cascade, resulting in the cleavage of hundreds of proteins leading to demolition of the cell. The death receptor pathway is activated by ligation of members of the tumour necrosis factor receptor (TNFR) superfamily that have an intracellular death domain by their respective ligands (e.g. FAS activated by FAS ligand) [24]. This promotes the formation of an intracellular death inducing signalling complex, resulting in the activation of caspase-8 and the downstream effector caspases (caspases-3 and -7) (Figs. 1 and 2) [25]. The death receptor pathway can connect to the intrinsic apoptotic pathway through caspase-8-mediated proteolytic activation of the pro-apoptotic BH3-only protein BID (Fig. 1) [24].

Amyotrophic lateral sclerosis (ALS) is a progressive adult-onset form of MND caused by the aberrant death of motor neurons in the cerebral cortex and spinal cord. The resulting motor neuron loss leads to muscle atrophy and weakness, muscle twitches, spasticity and typically death from respiratory failure [26]. Various molecular processes have been associated with this pathology, including oxidative stress, excitotoxicity and mitochondrial dysfunction [27]. Mutations in superoxide dismutase 1 (SOD1) account for ~20% of familial ALS cases [28]. A direct role for SOD1 in regulating the apoptotic machinery was proposed based on reports that on the surface of mitochondria in cells from the spinal cord mutant SOD1 was bound to BCL-2, with the contention that this would inhibit this anti-apoptotic protein [29]. Moreover, changes in the expression of certain BCL-2 family members and caspases have been observed in the spinal cord of transgenic mice expressing mutant SOD1 and from humans affected with ALS [30]. Abnormally reduced levels of anti-apoptotic BCL-2 have been reported in the SOD1^G93A^ mouse model [31, 32] as well as increased expression of the apoptosis effector BAX in spinal cord motor neurons of ALS patients [33]. Abnormally high mRNA levels of the BH3-only protein BIM were found in the spinal cord of post-symptomatic SOD1^G85R^ transgenic mice and, importantly, BIM deficiency extended the lifespan of SOD1^G93A^ mutant mice [34]. The onset of symptoms and terminal illness were also delayed in SOD1^G93A^ mice by administration of the broad spectrum caspase inhibitor zVAD-fmk [35], overexpression of BCL-2 [36] or the absence of the apoptosis effectors BAX and BAK [37].

AD is the leading cause of dementia. Hallmark pathological features of AD include the accumulation of amyloid-β-containing neuritic plaques derived from aggregates produced by sequential cleavages of the amyloid precursor protein (APP), neurofibrillary tangles and dystrophic neurites containing hyperphosphorylated tau [38]. Evidence for a role of apoptosis in neuronal cell death in AD is limited. However, based on immuno-histochemical staining of neurons, it has been proposed that intracellular Aβ can induce apoptosis through p53-dependent transcriptional upregulation of BAX [39, 40] and reductions in BCL-2 and BCL-XL [41]. It was also reported that caspase activation and their cleavage of APP are associated with synapse loss [42–44].

HD is characterised by progressive motor, behavioural and cognitive decline. This is driven by expanded CAG repeats in the *HTT* gene that encodes huntingtin. Observations in mouse models of HD include increased levels of pro-apoptotic BIM and BAX in brain lysates at late stages of the disease [45, 46]. Notably, loss of one allele of *Bim* significantly attenuated the accumulation of mHTT (mutant Huntington protein), neuronal death and disease-associated phenotypes [47]. BAX expression in the brain was reported to be maximal in grade 2 and 3 HD brains [48].

Degeneration of the dopaminergic neurons in the substantia nigra is the cause of motor dysfunction in PD that is characterised by a resting tremor as well as abnormal posture and gait. In PD, the predominant mechanism of neuronal death is thought to be via the intrinsic apoptosis pathway in dopaminergic neurons. Inherited forms of PD are associated with mutations in genes associated with mitochondrial function, such as *PRKN*, *LRRK2*, *PINK1* and *PARK7* [49]. The functions of the corresponding proteins may intersect with components of the intrinsic apoptosis pathway given that they are both located on the outer mitochondrial membrane. Indeed, Parkin was shown to suppress apoptosis by ubiquitinating BAK, thereby reducing its oligomerisation and apoptotic activity. PD-associated Parkin mutants had reduced ability to ubiquitinate BAK, suggesting that this would cause an increase in BAK-mediated apoptosis [50]. Caspase inhibitors were shown to be neuroprotective in in vitro models of PD [51]. Examination of brain tissue from PD patients revealed abnormally increased levels of active caspase-3 and BAX [52] as well as significant reductions in BCL-2, which correlated inversely with disease duration and severity [53]. These findings suggest that aberrant activation of the intrinsic apoptotic pathway may contribute to or even be a major driver of neuronal death in PD.

GBM is the most common form of brain cancer in humans with a poor prognosis (5-year survival of only 5%) that is largely due to its invasiveness. GBM cells are resistant to apoptotic stimuli and it is thought that this contributes to the failure of conventional standard of care treatment in this disease. Human glioblastoma cells were shown to express higher levels of BCL-2 and BCL-XL compared to non-neoplastic glial cells [54]. RNA interference mediated reduction in BCL-2 or BCL-XL was able to kill glioblastoma cells in culture, and this death was caspase dependant [54]. Furthermore, high levels of BCL-XL have been reported to be associated with rapid progression and poor survival of glioblastoma patients and BCL-XL has therefore been proposed as a marker of therapy resistance in this malignancy [55].

### Necroptosis

Necroptosis is a lytic form of PCD that can drive inflammation. Necroptosis can be induced by the stimulation of TNFR1, TLRs and certain other receptors when the activity of caspase-8 is blocked by pharmacological agents or viral inhibitors [56]. This process involves receptor-interacting serine/threonine protein kinase 1 (RIPK1), which is activated by autophosphorylation [57]. This enables RIPK1 to activate the kinase RIPK3 within a cytoplasmic high molecular weight complex termed the necrosome. RIPK3 then phosphorylates and thereby activates the pseudo-kinase MLKL, the terminal effector of necroptosis that causes lysis of the plasma membrane [58, 59]. This facilitates release of damage-associated molecular patterns (DAMPs), and in the case of infected cells also pathogen-associated molecular patterns (PAMPs) [60], driving an inflammatory response (Fig. 1).

In ALS, necroptosis was reported to be dispensable for motor neuron degeneration, based on the observation that the absence of MLKL did not affect disease onset, progression and survival in SOD1^G93A^ mutant mice [61]. In contrast, there was evidence of necroptosis in post-mortem examination of brains from human AD patients, with abundant expression of MLKL compared to brains from healthy controls. Moreover, necroptosis was postulated to exacerbate cognitive deficits in the APP/PS1 mouse model of AD, since treatment with the RIPK1 inhibitor necrostatin-1 reduced neuronal death, attenuated the formation of insoluble Aβ plaques and hyperphosphorylated tau in the cortex and hippocampus and ameliorated cognitive impairment [62, 63]. It is, however, noteworthy that in addition to inducing necroptosis, RIPK1 (and RIPK3) are also involved in activating caspase-8 mediated apoptosis and the production of cytokines and chemokines [64]. It remains unclear inhibition of which of these processes by necrostatin-1 reduced pathology in the APP/PS1 mice. A sub-type of disease-associated microglia has been implicated in promoting the formation of Aβ plaques in AD. A study showed that RIPK1 may promote this behaviour of microglia, thereby triggering inflammation and contributing to pathogenesis [65]. Pharmacological inhibition or genetic ablation of RIPK1 in a mouse model of AD reduced amyloid burden, the levels of inflammatory cytokines and memory deficits [65]. Therefore, RIPK1 is considered a promising target for therapeutic intervention in this disease. In preclinical models of PD, genetic ablation of MLKL or RIPK3 or pharmacological inhibition of RIPK1 exerted neuroprotective effects, with decreased dopaminergic neuron degeneration and improved motor performance. Moreover, phosphorylated (i.e. activated) MLKL was found in post-mortem brain biopsies of human PD patients [66]. In a tissue culture model of PD, treatment with a RIPK1 inhibitor protected iPSC-derived neural cells from PD patients harbouring mutations in the optic atrophy type 1 *(OPA1)* gene from death and reduced oxidative stress [67].

Evidence of a role of necroptosis in the pathogenesis of HD is limited. One study reported that in the R6/2 transgenic mouse model of HD, in which *exon* 1 of a mutated human *HTT* gene is expressed and driven by the human huntingtin promoter [68], treatment with Necrostatin-1 ameliorated symptoms and delayed disease progression, thus identifying a role for RIPK1 in disease progression [69]. However, to date there are no reports on the expression of necroptosis signalling proteins in post-mortem samples from HD patients. Overall, these studies provide evidence that necroptosis may play a role in disease pathogenesis and that inducers of necroptosis, such as RIPK1, may constitute promising ‘druggable’ targets for this neurodegenerative disease.

Stroke constitutes the second leading cause of mortality after ischaemic heart disease. It is caused by insufficient blood flow to the brain, triggering a cascade of pathological responses, including inflammation, ROS production and protein misfolding [70]. In animal models of stroke, such as hypoxia-ischaemia, oxygen-glucose deprivation and collagenase-induced intracerebral haemorrhage, treatment with the RIPK1 inhibitor necrostatin-1 or genetic ablation of proteins critical for necroptosis improved neurological function and attenuated neuronal cell death post brain injury [71–73]. While some studies have indicated that necroptosis plays a role in the pathogenesis of stroke and treatments targeting necroptosis signalling proteins have proved to be neuroprotective in various animal models [70], research into the clinical utility of necroptosis inhibitors in patients is lacking and this area warrants further investigation.

In cancer, there are contradictory reports claiming that necroptosis can either promote or inhibit tumour growth [74], perhaps depending on the type of cancer or whether necroptosis occurs in the malignant cells or in cells of the tumour microenvironment. In head and neck squamous cell carcinoma, RIPK1 expression is downregulated compared to healthy tissues [75], whereas in lung cancer patients and mouse models of lung cancer, RIPK1 expression is markedly elevated in the tumour tissue [76]. In a study of GBM patients, ~30% of tumours exhibited high levels of RIPK1 expression [77] and this correlated with adverse prognosis. Amongst patients with lower grade gliomas, those with higher RIPK3 expression levels had poorer prognosis [78]. In another study, upregulation of MLKL in GBM patients was associated with an unfavourable prognosis [79]. It therefore appears that increased expression of RIPK1, RIPK3 and MLKL may promote tumour growth. This may be linked to reports that necroptosis in cells of the tumour microenvironment drives angiogenesis and inflammation, which can promote cancer cell proliferation and metastasis [80]. With the observations that glioblastoma cells are highly resistant to apoptosis, activation of necroptosis or alternate mechanisms of PCD, may represent promising avenues to explore for cancer therapy. Activators of RIPK1, RIPK3 and MLKL may constitute possible approaches, although the safety of such strategies will need to be established.

### Cell death associated with autophagy

Autophagy is a highly conserved process for the degradation of macro-molecular structures and even entire organelles that plays critical roles in cellular and tissue homoeostasis [81]. This process is important for regulating the cytoplasmic turnover of proteins and entire organelles. A myriad of stimuli can enhance autophagy, including nutrient deprivation, oxidative stress and protein aggregates. In these settings, autophagy reduces cell stress and provides cells with metabolites for repair, survival and growth. Autophagy can be differentiated into three subtypes: macro-autophagy, micro-autophagy and chaperone-mediated autophagy; for a comprehensive review see ref. [82]. Each of these processes is distinct, however, they all converge upon lysosomes for cargo degradation and recycling of intracellular content. Although autophagy is often used to promote cell survival, in certain settings, such as the involution of salivary glands during Drosophila development [83], autophagy is associated with cell killing.

A hallmark of neurodegenerative diseases includes the accumulation of proteinaceous aggregates and ubiquitinated inclusion bodies and they are thought to be involved in the aetiology of these diseases. Aberrant autophagy is a feature of several neurological diseases. In ALS, mutations in autophagy-related genes, including *SQSTM1*, *OPTN*, *TBK1*, *VCP* and *C9ORF72*, are associated with familial forms of the disease. The accumulation of autophagosomes in the cytoplasm of spinal cord neurons of ALS patients has been reported [84], as well as an increase in the formation of autophagosomes in SOD1 mutant transgenic mice [85]. In the SOD1^G93A^ mouse model of ALS loss of *Atg7* accelerated neuromuscular denervation and the onset of hindlimb tremor at the early-symptomatic stage of the disease. However, at late stages of disease, autophagy had an adverse role, where loss of *Atg7* slowed disease progression and increased the lifespan of the mice [86].

The accumulation of autophagosomes in neurons is a conspicuous feature of AD in both animal models and patients with observations of increased Aβ generation and accumulation in lysosomes in cells with defects in autophagy. This suggests that the turnover of Aβ is regulated in part by autophagy [87]. Microarray profiling of hippocampal CA1 pyramidal neurons from post-mortem brain tissues from AD patients or controls revealed high expression of autophagy-related genes in early stages of AD. This correlated with increased levels of autophagosome components, increased LC3-positive puncta and defective clearance of autophagic substrates by lysosomes in CA1 pyramidal neurons [88]. Therefore, enhancement of autophagy may be a promising area of investigation for achieving neuroprotective outcomes in AD.

Defects in autophagy are associated with several molecular mechanisms underpinning PD, and several *ATG* genes were shown to be aberrant in PD [89]. Accordingly, dysregulated autophagy has been identified in brain tissues from PD patients and in PD animal models, suggesting that autophagy plays a role in disease pathogenesis [90].

It is a similar scenario in HD, where accumulation of the huntingtin protein (HTT) is associated with attenuated autophagy [91]. Notably, enhanced autophagy by inhibition of mammalian target of rapamycin signalling was found to enhance the clearance of HTT aggregates and reduced toxicity in Drosophila and mouse models of HD [92]. Paradoxically, ablation of p62, an autophagy receptor, significantly attenuated the formation of nuclear inclusions and motor deficits and prolonged lifespan in a mouse model of HD [93]. These reports exemplify the complexity of targeting autophagy signalling in HD (and possibly other neurological diseases). Thus, substantial additional work is required to arrive at a better understanding of the role of autophagy in HD pathogenesis, so that this knowledge may be used to develop new treatments for HD that are based on manipulating this process.

The role of autophagy in GBM is controversial, with some reports claiming that it suppresses tumour growth whereas others stated that it promotes tumour growth. In U343 glioma cells, autophagy was shown to trigger cell senescence [94] and this also enhanced TMZ-induced senescence in glioma cells [95]. Triggering autophagy was reported to inhibit GBM cell migration and invasiveness, reversing the epithelial-mesenchymal transition [96]. Conversely, autophagy was also reported to exert positive effects on tumours, increasing their proliferative potential and elevated expression of p62 was correlated with poorer survival in GBM patients [97]. Perturbations in EGFR, PTEN and AKT, which are frequently mutated in GBM, have been reported to impact the regulation of autophagy [98]. Autophagy was also shown to promote survival of GBM cells [99] and to facilitate metastasis [100]. Given the ongoing controversy about the role of autophagy in glioblastoma (and many other cancers for that matter [101]), further interrogation of this pathway is warranted if it should be harnessed for therapeutic intervention in this disease.

### Ferroptosis

First coined in 2012 [102], ferroptosis refers to a form of iron-dependent necrotic PCD. The final executor in ferroptosis is overwhelming lipid peroxidation causing complete cell failure. Although ferroptosis exhibits many features of what was previously commonly called oxidative stress-induced cell death, there are many aspects that distinguish it as a distinct form of cell death. For instance, ferroptosis is morphologically and functionally distinct from ‘generic’ oxidative stress, such as hydrogen peroxide-induced necrosis [102]. Many molecular components of ferroptosis have been identified, including ACSL4 and LPCAT3 that generate the membrane lipids susceptible to peroxidation [103, 104], and the glutamate-cystine antiporter system xCT required to supply cysteine to the cell. Critical to prevent lipid peroxidation are endogenous mechanisms including glutathione peroxidase 4 (GPX4; [105] and ferroptosis suppressor protein 1 (formerly AIFM2) [106, 107] which use glutathione and ubiquinone, respectively, as reducing substrates, and tetrahydrobiopterin synthesised by GTP cyclohydrolase-1 [108]. Ferroptosis inducers include inhibitors of GPX4 (RSL3, ML210, ML162, FIN56, FINO2), disruption of glutathione synthesis (buthionine sulfoximine), disruption of cysteine supply via inhibition of system xCT (erastin, sorafenib, sulfasalazine, glutamate), iron and iron-disrupting stimuli. Endogenous inhibitors of ferroptosis include glutathione, ubiquinone, vitamin E and selenium. Exogenously applied ferroptosis inhibitors include radical trapping antioxidants (RTAs; ferrostatin-1, liproxstatin-1), inhibition of lipoxygenases (in absence of RTA activity, see below) and iron chelators (deferoxamine, deferiprone). The selectivity and potency of RTAs illustrates the unequivocal role of lipid peroxidation in ferroptosis.

Many small molecule inhibitors are redox active and exhibit significant potential to directly inhibit lipid peroxidation [109]. This is problematic when attempting to delineate additional molecular aspects of ferroptosis as such inhibitors may directly inhibit lipid peroxidation independent of interactions with their intended targets. For instance, many lipoxygenase inhibitors are redox active; this includes zileuton, NGDA, baicalein and PD146176 [109]. Therefore, the use of redox active molecules should be coupled with complementary experimental approaches in order to permit clear interpretation of the findings. Furthermore, when a redox active small molecule exhibits efficacy in a given model of disease, this may be due to inhibition of ferroptosis rather than (or in addition) to their intended action. In light of the increasing evidence for a role of ferroptosis across many conditions, this calls for a re-evaluation of previous studies involving inhibitors that are redox active and inhibit lipid peroxidation.

Specific evidence for a role of ferroptosis in a disease setting is difficult to establish. Almost all neurodegenerative diseases appear to exhibit lipid peroxidation. Likewise, dysregulated iron homoeostasis and diminished glutathione are also common features of neurodegeneration. Perhaps the best evidence comes from the protection afforded by ferroptosis inhibitors in animal models of disease and ultimately in human clinical trials. The canonical RTA ferroptosis inhibitors liproxstatin-1 and ferrostatin-1 have been reported to exhibit efficacy in mouse models of stroke [110], PD [111] and HD slice culture assays [112]. Vitamin E prevents the rapid death of neurons in conditional neuronal *Gpx4* knockout mice [113, 114], whereas overexpression of GPX4 protects against intracerebral haemorrhage in rats [115].

Several ferroptosis inhibitors have been assessed in the clinic. In a phase II clinic trial for AD, the iron chelator deferoxamine significantly reduced the rate of cognitive decline in patients [116]. Surprisingly, this study is yet to be replicated after 30 years, although nasal formulations of deferoxamine are showing promise in animal models of AD [117, 118].

More recently, therapeutic strategies targeting iron have focused on the iron chelator deferiprone. Similar to deferoxamine, deferiprone inhibits ferroptosis in vitro, and exhibits efficacy in mouse models of AD, PD and ALS [119–121]. In two phase II trials for PD, deferiprone significantly impacted brain iron levels and either significantly delayed or trended towards slowing progression of symptoms as measured by the UPDRS [117, 121]. A third phase II trial with 140 participants has been completed but is yet to report, while a fourth phase II trial with 372 participants is currently underway. Deferiprone is also currently under clinical investigation for AD and ALS in two large phase II trials.

Cu^II^(atsm) strongly inhibits ferroptosis induced by RSL3 or erastin in neural cells in vitro and in a cell-free lipid peroxidation system [122]. Cu^II^(atsm) has been extensively investigated (and independently validated) in preclinical animal models and exhibits efficacy in multiple models of ALS, PD and stroke (for review see [123]). Phase I clinical trials for ALS and PD have been completed with encouraging results [124]. A phase II trial with 80 participants is currently ongoing, as well as extension trials for ALS patients from both trials.

Ferroptosis-related gene expression is associated with diagnostic and prognostic factors in glioma [125]. Many anti-cancer drugs appear to target and enhance ferroptosis to kill glioma cells, including withaferin A [126], dihydroartemisinin [127] and ibuprofen [128]. Inducing ferroptosis is thought to enhance effects of ‘traditional’ anti-cancer treatments that trigger other cell death pathways, mostly apoptosis. Feeding glioblastoma bearing mice or rats with iron enhanced the impact of radiation therapy [129, 130]. Moreover, inhibition of xCT by erastin or sulfasalazine potentiated the efficacy of temozolomide [131, 132]. Coatomer Protein Complex, Subunit Zeta 1 (COPZ1) is associated with increased tumour grade. Inhibition of COPZ1 using RNA interference inhibited tumour growth and enhanced survival in mice by increasing intracellular iron by enhancing ferritinophagy [133]. Conversely, glioblastoma cell necrosis that was reported to be driven by neutrophil-triggered ferroptosis was shown to be associated with worsened outcomes [134]. Overall, it appears that the effects of ferroptosis are dependent on many factors. Ferroptosis may kill cancer cells, however, it is not immune-silent raising the issue of what impact this may have on surrounding healthy tissues.

### Pyroptosis

Pyroptosis is an inflammatory form of PCD involving activation of caspase-1 by inflammasomes. Caspase-1 proteolytically processes pro-IL-1β and pro-IL-18 into the mature inflammatory cytokines IL-1β and IL-18, respectively. Gasdermin D (GSDMD) is the critical executioner of pyroptosis [135]. Caspase-1 cleaves GSDMD and its N-terminal fragment assembles into a plasma membrane pore [136]. This is required for release of bio-active IL-1β and IL-18 as well as other cellular contents, and for the killing of the cell. Historically, pyroptosis was often thought to be a monocyte-specific form of apoptosis, as it exhibits a plasma membrane-blebbing morphology. However, the recent discovery of GSDMD and its pore-formation activity has redefined pyroptosis as a necrotic form of cell death.

Evidence for pyroptosis (accompanied by inflammasome activation and elevated IL-1β and IL-18) has been reported for many neurodegenerative diseases, including AD, PD, ALS, HD, multiple sclerosis, stroke and traumatic brain injury [137]. Inflammasome activation and pyroptosis have been found in microglia and oligodendrocytes in an animal model of multiple sclerosis, in which pathology was diminished by caspase-1 inhibition [138].

### Necrosis: unregulated cell death

Necrosis is classically considered an unprogrammed and unregulated cell death process that is characterised by cell swelling, loss of membrane integrity, ‘spillage’ of intracellular contents (DAMPs and PAMPs) into the extracellular environment and dissipation of ion gradients, overall triggering an inflammatory response. Necrosis can occur due to overwhelming stimuli from outside the cell, such as hypoxia, freezing or burning, certain pathogens, physico-chemical stresses (e.g. H_2_O_2_), ischaemia-reperfusion and calcium overload [139]. Early events in necrosis include an increase in intracellular Ca^2+^ concentration and generation of reactive oxygen species culminating in events that result in irreversible cell injury. However, unlike necroptosis, necrosis lacks a defined core cellular signalling machinery but Ninj1 has recently been identified as being critical for the rupture of the plasma membrane [140]. Of note, Ninj1 is also critical for the final rupture of the plasma membrane that occurs during necroptosis, pyroptosis and the secondary necrosis that is seen when cells undergoing apoptosis are not engulfed by neighbouring phagocytes. Necrosis is observed in many pathological conditions, including myocardial infarction, stroke, several neurodegenerative diseases and in certain cancers, where factors released from necrotic malignant cells are likely to impact the tumour microenvironment [14, 141].

Since necrosis is an unprogrammed form of cell death, it has been proposed that the necrotic pathology associated with neurological diseases emanates from an interplay of finely tuned programmed necrosis cascades, such as necroptosis, ferroptosis and pyroptosis, and that this is a driver of the neurological diseases (Table 1). It is therefore anticipated that targeting the necrotic PCD pathways may provide novel opportunities for therapeutic intervention which is discussed below. Of note, it remains a distinct possibility that in neurodegenerative diseases at least some aspects of tissue damage, such as the immediate insult from the occlusion of blood vessels in stroke, is actually caused by unregulated (non-programmed) necrosis rather than by any of the programmed necrotic cell death pathways. Such tissue damage may only be alleviated by preventing or reducing the insult causing the patholoogy in the first place (e.g. increasing perfusion during stroke). Nonetheless, most of the secondary (and possibly tertiary) tissue destruction in neurodegenerative diseases may well be caused by an interplay of apoptosis and the necrotic PCD pathways. Therefore, inhibitors of MLKL, GSDMD and BAX/BAK, the effectors of necroptosis, pyroptosis or apoptosis, respectively, may allow improved outcomes for patients with these diseases [142]. It appears likely that such agents will need to be used in combination due to the ability of cells to engage another PCD process when the one they would normally undergo is blocked [143, 144].

### Therapeutic implications

The development and study of mouse models (e.g. genetic modifications or treatment with toxic insults) mimicking neurological diseases has led to an understanding of key regulators of the different cell death signalling pathways and their relevance in disease pathogenesis. Even though aberrant apoptotic cell death and expression of key pro-apoptotic proteins are associated with neurodegenerative diseases, targeting apoptosis in vivo has so far proved disappointing. The clinical potential of Minocycline, a second-generation tetracycline was tested in various preclinical mouse models of neurodegenerative diseases, such as ones for ALS, PD and HD. Minocycline blocks the release of cytochrome c from mitochondria, inhibiting this step in apoptosis, and was reported to upregulate the expression of anti-apoptotic BCL-2, also exhibiting anti-inflammatory and antioxidant effects, displaying effective neuroprotective outcomes in preclinical mouse models [145]. However, a recent randomised clinical trial reported that Minocycline was ineffective and failed to delay disease progression in patients with mild AD over a 24 month period [146]. The reason for this is likely that MOMP and loss of ATP production in mitochondria will still occur despite treatment with this agent. Thus, cells exposed to Minocycline would still be ‘functionally dead’.

Pharmacological inhibition or genetic ablation of RIPK1 has produced neuroprotective outcomes in preclinical models of AD, PD and HD. The use of the blood brain barrier-penetrant RIPK1 inhibitor DNL747 was tested in a clinical trial for AD and ALS, even though necroptosis was reported to be dispensable for the latter. DNL747 progressed through phase I trials. However, the trial was then halted in favour of its successor compound, DNL788, which is anticipated to be superior in achieving neuroprotective outcomes [147]. The efficacy of the combination of sodium phenylbuturate and Taurursodiol was recently reported in a randomised, double-blind trial for ALS. Taurursodiol was reported to exert anti-apoptotic properties, inhibiting the translocation of the apoptosis effector BAX to mitochondrial membranes, while sodium phenylbutrate, a histone deacetylase inhibitor can ameliorate toxicity from endoplasmic reticulum stress, thereby promoting cell survival. Measurement of drug impact included examination of functional decline, which was reported to be slower compared to the placebo treated control subjects, when assessed over a 24-week period [148].

Bcl2l12, a protein containing a BH2 domain (but none of the other BCL-2 family homology (BH) regions) was reported to drive the development of GBM by interacting with certain members of the BCL-2 protein family and inhibiting the activation of caspases-3 and -7, thereby inhibiting mitochondrial-induced apoptosis [149]. An early phase clinical trial (NCT03020017) in GBM involved evaluating the efficacy of utilising spherical nucleic acid gold nanoparticles composed of siRNAs targeting Bcl2l12 (NU-0129). The nanoparticles can cross the blood brain barrier; therefore, it is anticipated that NU-0129 will penetrate into the tumour tissue and will be able to inhibit the growth of GBM.

Regulators and effectors of the different cell death pathways remain attractive therapeutic targets that may form the basis for translational work that will hopefully lead to improvements for patients with these diseases. Given that the aetiology of neurological diseases is complex, where multiple cell death mechanisms often in conjunction with other cellular processes drive pathology, it appears likely that effective therapies will comprise inhibitors of more than one cell death programme plus inhibitors of additional cellular processes. Table 2 displays some key targets within cell death pathways which have advanced to clinical trials.

**Table 2:** Candidate drugs targeting cell death pathways in neurological diseases.

Cell death pathway	Drug and mechanism of action	Disease	Clinical trial phase (number of participants) and outcome	References and/or clinical trial registration number
Apoptosis	Carboplatin, DNA damaging agent triggering apoptosis. Evaluated in combination with Bevacizumab (VEGF inhibitor).	GBM	Phase II (122), patients on carboplatin + Bevacizumab; more toxicity without additional clinical benefit compared to placebo.	ACTRN12610000915055 [153]
	Olaparib, PARP inhibitor, sensitizes GBM cells to death receptor-mediated apoptosis induced by TRAIL. These agents all induce apoptosis in malignant cells	GBM	PhaseI/IIa (79), Evaluating the therapeutic potential of Olaparib in combination with TMZ and radiation.	NCT03212742 [154]
Necroptosis	DNL747; RIPK1 inhibitor	ALS	Phase I (15)	NCT03757351
		AD	Phase I (16)	NCT03757325
Autophagy	Rapamycin; Autophagy enhancer, mTOR inhibitor.	ALS	Phase II (63)	[155]
	Resveratrol; autophagy enhancer	AD	Phase III (27), No significant changes in Alzheimer’s Disease Assessment Scale.	NCT00678431 [156]
			Phase II (119), no effects of drug treatment on plasma Aβ42, CSF Aβ42, CSF tau, CSF phospho-tau and hippocampal volume.	NCT01504854 [157]
	CQ; autophagy inhibitor, and chemoradiation with Temozolomide (alkylating agent).	GBM	Phase III (30), Median survival increased in patients receiving CQ + standard of care treatment.	NCT00224978 [158]
Ferroptosis	Deferiprone; iron chelator	PD	Phase II (40) Stabilised brain iron and slowed disease progression (UPDRS).	NCT00943748 [121]
			Phase II (22) Deferiprone therapy reduced brain iron content and trended towards improved motor -UPDRS scores and quality of life, but was not significant.	NCT01*5*39837 [159]
			Phase II (140) Yet to report.	NCT02728843
			Phase II (372) Ongoing	NCT02655315
		AD	Phase II (171) Ongoing	NCT03234686
		ALS	Phase II (23) Decreased iron in spinal cord and motor cortex. Slower disease progression (ALSFRS-R) and weight loss.	NCT02164253 [120]
			Phase II (240) Ongoing	NCT03293069
		FRDA	Phase II 2010 (80)	NCT00530127
			Phase II 2011 (36)	NCT00897221
	Edaravone; radical scavenger	ALS	FDA-approved	
	Cu^II^(atsm); radical scavenger	PD	Phase I (31) Not reported	NCT03204929
		ALS	Phase I (50)	NCT02870634 [124]
			Phase I (28) Ongoing	NCT03136809
			Phase II (80) Ongoing	NCT04082832
			Phase II (70) Ongoing	NCT04313166

*VEGF* vascular endothelial growth factor, *PARP* poly ADP-ribose polymerase, *TRAIL* tumour necrosis factor-related apoptosis-inducing ligand, *mTOR* mammalian target of rapamycin, *CQ* chloroquine, *FRDA* Friedreich’s ataxia, *ACTRN* Australian clinical trial registration number, *NCT* clinical trials.gov identifier, *CSF* cerebrospinal fluid, *UPDRS* Unified Parkinson’s Disease Rating Scale, *ALSFRS-R* Revised Amyotrophic Lateral Sclerosis Functional Rating Scale, *FDA* United States Food and Drug Administration.

## Conclusions and perspectives

Many diseases of the brain are associated with defects in one or several processes of PCD: either aberrant killing of cells that should survive in neurodegenerative disorders or aberrant survival of cells that should die during the development and therapy of brain cancers. In most neurodegenerative diseases it has not yet been unequivocally defined whether these defects in cell death are a true cause or at least critical contributor to disease or simply a consequence of some insult to the tissue where even effective blockade of this cell death would not offer improved therapy. In the case of brain cancer, as with all types of cancer, effective delivery of potent inducers of any type of cell death would be expected to cause tumour shrinkage, of course with the proviso that it will be safe, i.e. tolerable to vital tissues. We contend that much additional research, both basic work in animal models and studies with patient material, is needed to garner a more detailed understanding of the roles of the different processes of cell death in diseases of the brain, so that this knowledge can be harnessed to develop truly transformative advances in their treatment.

### Facts

- Neurodegenerative diseases are associated with aberrant neuronal cell death, but the processes leading to such cell death and the mode of cell death still remain unclear.
- Glioblastoma cells are highly resistant to apoptosis; therefore, activation of alternate mechanisms of PCD may represent promising avenues to explore for therapy of this cancer.
- With the brain’s complex cellular and architectual diversity, it is not unreasonable to predict that many forms of cell death may be occurring simultaneously in disease states. Indeed, evidence indicates this is likely the case, with various inhibitors targeting many forms of cell death having beneficial impact in the same disease model.
- Given there are perturbations in more than one PCD signalling pathway and also unprogrammed necrosis in disease pathogenesis, effective therapies will need to comprise inhibitors of more than one type of cell death.

### Open questions

- Is a defect in a PCD signalling pathway a direct cause or a critical contributor to disease or simply a consequence of some insult to the tissue where even effective blockade of cell death would not offer improved outcome?
- Programmed pathways of lytic cell death are inherently more immunogenic than apoptosis. Will inhibition of lytic PCD pathways be more effective in the treatment of neurodegenerative diseases than blocking apoptosis because the former induces a pro-inflammatory state? Are neurons more susceptible to death in an inflammatory state?
- The ferroptosis PCD pathway is not immune-silent. What impact would this have on surrounding healthy tissues when trying to exploit inducers of ferroptosis for the treatment of brain cancers?
